# OVTwin: a GPU-accelerated digital twin for simulating combination chemotherapy in epithelial ovarian cancer

**DOI:** 10.1093/bib/bbaf631.008

**Published:** 2025-12-12

**Authors:** Xinyue Chen, Jiale Qin, Fenglan Pan, Zhiang Chen, Xiaodong Wu, Chen Li

**Affiliations:** Department of Human Genetics, Department of Ultrasound, Department of Gynecological Oncology and Women's Hospital, Zhejiang University School of Medicine, Hangzhou, 310058, China; Zhejiang University-University of Edinburgh Institute (ZJU-UoE Institute), Zhejiang University, Haining, 314400, Zhejiang, China; Department of Human Genetics, Department of Ultrasound, Department of Gynecological Oncology and Women's Hospital, Zhejiang University School of Medicine, Hangzhou, 310058, China; Department of Human Genetics, Department of Ultrasound, Department of Gynecological Oncology and Women's Hospital, Zhejiang University School of Medicine, Hangzhou, 310058, China; Zhejiang University-University of Edinburgh Institute (ZJU-UoE Institute), Zhejiang University, Haining, 314400, Zhejiang, China; Department of Human Genetics, Department of Ultrasound, Department of Gynecological Oncology and Women's Hospital, Zhejiang University School of Medicine, Hangzhou, 310058, China; Department of Human Genetics, Department of Ultrasound, Department of Gynecological Oncology and Women's Hospital, Zhejiang University School of Medicine, Hangzhou, 310058, China; Department of Human Genetics, Department of Ultrasound, Department of Gynecological Oncology and Women's Hospital, Zhejiang University School of Medicine, Hangzhou, 310058, China; Zhejiang University-University of Edinburgh Institute (ZJU-UoE Institute), Zhejiang University, Haining, 314400, Zhejiang, China

## Abstract

**Background:**

Ovarian cancer is a highly lethal gynecological malignancy, with most cases diagnosed at advanced stages. First-line therapy combines cytoreductive surgery with paclitaxel-carboplatin chemotherapy, yet responses differ markedly between patients. Most experimental systems are limited in both scale and duration, and few computational models can reproduce full chemotherapy courses. Understanding how drug exposure and tumor response drive these divergent outcomes remains difficult. New approaches are needed to study tumor-drug interactions over clinically relevant timescales and within sizeable 3D tumor spheroids.

**Method:**

We developed OVTwin, a GPU-accelerated digital twin framework, that models drug diffusion, cellular uptake, and cell fate dynamics in a three-dimensional ovarian tumor spheroid, and performs massively parallel simulations of the standard paclitaxel-carboplatin regimen over three chemotherapy cycles (63 days). The framework employs a fully GPU-parallelized architecture to efficiently run large-scale simulations and support exploration of multiple treatment settings.

**Result:**

OVTwin completed simulations of spheroids containing up to 500,000 cells within one hour. The model successfully reproduced the temporal dynamics of cell proliferation and apoptosis under the standard paclitaxel–carboplatin regimen. Additional simulations of paclitaxel-only, carboplatin-only, and untreated controls further confirmed that the combination regimen achieved the most effective tumor suppression, in line with clinical practice.

**Conclusion:**

By capturing the dynamics of tumor-drug interactions at cellular resolution and enabling rapid, large-scale simulations, OVTwin provides a framework for exploring patient-specific chemotherapy responses. Our findings demonstrate that GPU-accelerated digital twins can serve as an effective tool for investigating individualized chemotherapy strategies in ovarian cancer.

